# Low-Intensity Pulsed Ultrasound Alleviates Human Testicular Leydig Cell Senescence In Vitro

**DOI:** 10.3390/ijms24010418

**Published:** 2022-12-27

**Authors:** Sha Han, Jiaqiang Luo, Shuai Xu, Liangyu Zhao, Chencheng Yao, Junwei Xu, Ruhui Tian, Erlei Zhi, Yuhua Huang, Shujie Xia, Zheng Li, Peng Li

**Affiliations:** Department of Andrology, The Center for Men’s Health, Urologic Medical Center, Shanghai Key Laboratory of Reproductive Medicine, Shanghai General Hospital, Shanghai Jiao Tong University School of Medicine, Shanghai 200080, China

**Keywords:** LIPUS, Leydig cells, aging, testosterone

## Abstract

Aging has a significant negative impact on human testicular function; steroidogenesis is gradually impaired, and testosterone replacement therapy still has many risks. Low-intensity pulsed ultrasound (LIPUS) has been used as a novel non-invasive treatment for male erectile dysfunction and other fields, and has been shown to increase testosterone levels in animal models. Testosterone is synthesized and secreted by Leydig cells (LCs), and the serum testosterone level decreases after aging due to the LCs senescence. However, the effect of LIPUS on human senescent LCs has not been reported. In this study, human senescent LCs were isolated and stimulated with different energy intensities in vitro, and cell morphology, cell apoptosis, cell proliferation, cell senescence levels, lipid droplet number, testosterone and INSL3 secretion levels were tested and analyzed. Quantitative Polymerase Chain Reaction (QPCR) and Western Blot were performed to compare cell senescence characteristics and the expression profile of key pathways of testosterone secretion, and transcriptome analysis was performed to explore the signaling pathways of LCs alteration after LIPUS stimulation. It was safe and effective to stimulate LCs with the 75 mW/cm^2^ energy of LIPUS in vitro, which not only improved the senescence phenotype, but also effectively enhanced the secretory function of LCs in vitro, and increased the expression of key pathways of the testosterone synthesis pathway. These results suggest that LIPUS could be used as a novel treatment to human senescent LCs with decreased testosterone secretion levels in vitro.

## 1. Introduction

Testosterone synthesis has two peaks during fetal period and post-adolescence, and gradually declines with the aging of Leydig cells (LCs) after age 40 in males [1].Activated by luteinizing hormone (LH) signaling, Leydig cells produce the male hormone testosterone (T), critical for maintaining both spermatogenesis in the testes and the male sex characteristics of the body [2,3]. Moreover, increasing age has been found to be negatively associated with the dysregulation of Leydig cells in number, signaling, testosterone, secretory function and developmental identity [4,5]. However, there is currently no long-term effective treatment for the aging of Leydig cells.

Low-intensity pulsed ultrasound (LIPUS) is a low power ultrasound with a certain pulse waveform and is a new technique validated in different clinical settings, especially in orthopaedics and urology [6,7]. Ultrasound treatment has been proved to promote a significant increased serum testosterone level in prepubertal male rats, but the mechanism by which it affects testosterone levels remains unclear [8,9]. Nevertheless, Leydig cells have been successfully isolated in rats, and many in vitro studies have been reported [10,11,12]. Studies on human Leydig cells are limited to in vivo study and adult Leydig cells were only isolated in a few studies, and no further in vitro culture and intervention studies have been carried out [13]. The use of in vitro cell culture systems has been of central importance for research at the cellular and molecular levels. In addition, the key pathways and mechanisms of aging Leydig cells have been studied in vitro. Previous studies have verified the multiple biological effects of LIPUS on cells in vitro [14]. Thus, we hypothesize that LIPUS treatment can affect aging LCs functions in vitro. In this study, LIPUS was used to intervene in aging Leydig cells in vitro and evaluate the optimal stimulation energy intensity, and identify the key pathway of aging and provide a theoretical basis and in vitro experimental reference for aging treatments.

## 2. Results

### 2.1. Identification of LCs and Biosafety Assessment of LIPUS

**Figure 1:**
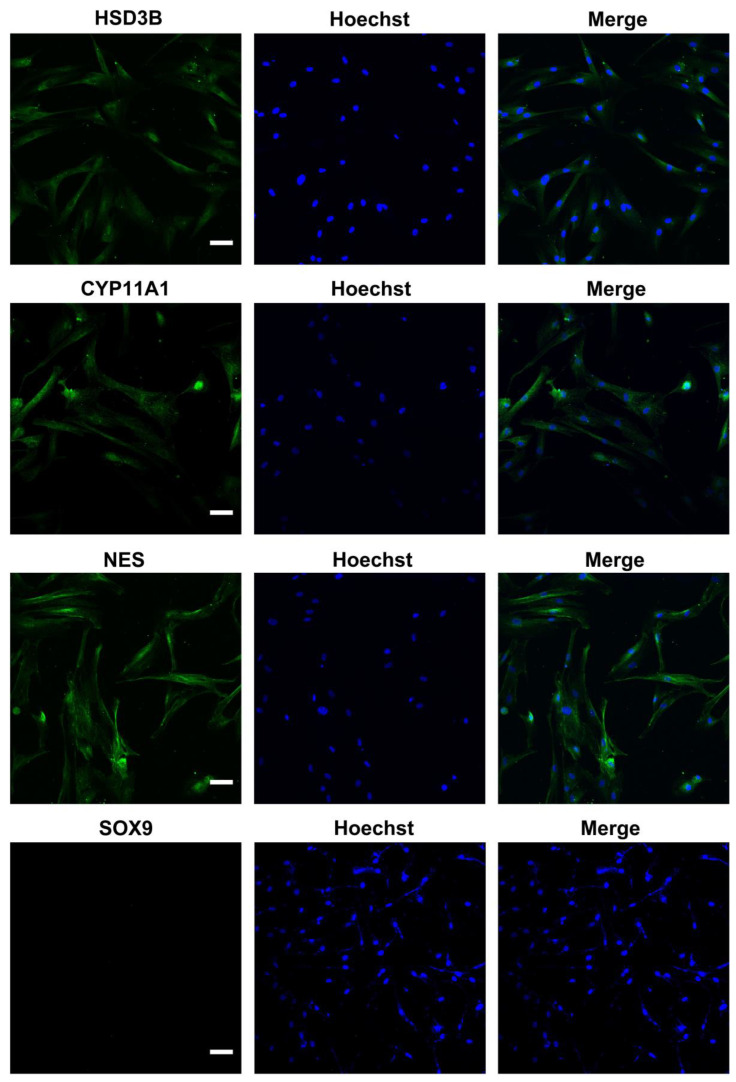
Identification of ALCs. Isolated cells were positive for LC markers (HSD3B, CYP11A1, NES) but negative for SC marker SOX9. The scale bar represents 100 μm.

**Figure 2:**
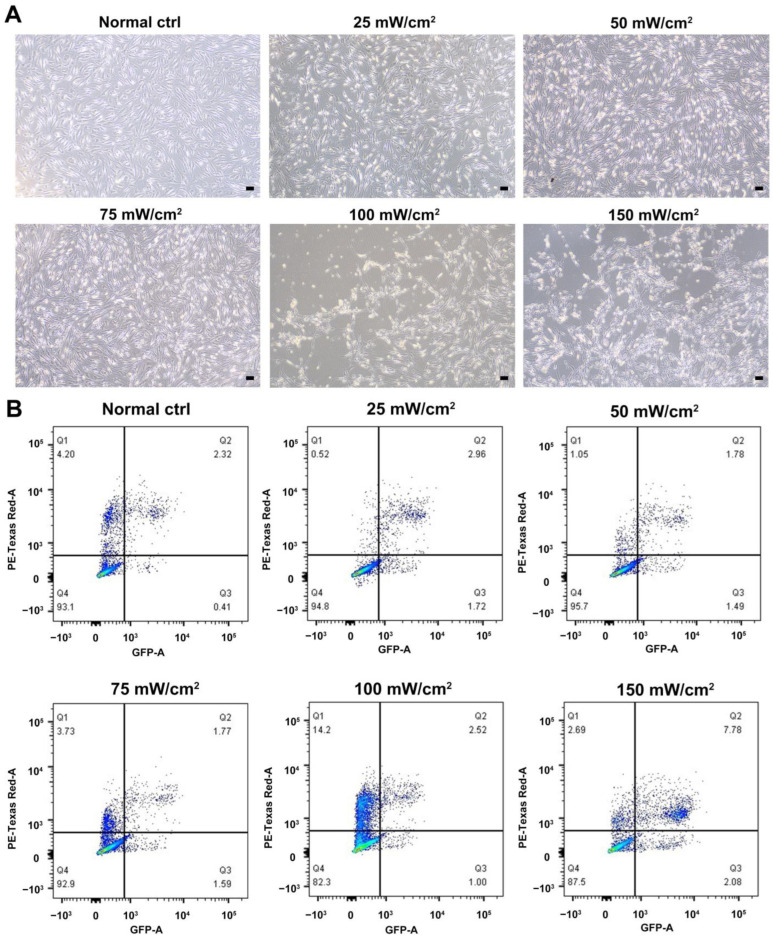
Biosafety assessment of LIPUS. LIPUS of different intensities was applied to LCs to evaluate its biosafety. The results of light microscope observation (**A**) and Annexin-V and PI staining and flow cytometry (**B**) demonstrated that 100 mW/cm^2^ and 150 mW/cm^2^ LIPUS will cause more apoptosis to LCs. The scale bar represents 100 μm.

We characterized the in vitro isolated Leydig cells, and the results demonstrated that LCs were positive for LC markers (HSD3B, CYP11A1, NES) but negative for SC marker SOX9 (Figure 1). Meanwhile, we compared the differences between young and aged LCs and found that aged LCs showed features such as increased b-gal ratio, reduced hormone secretory capacity and decreased transcript levels of testosterone secretion-related enzymes (Figure S1). LIPUS of different intensities was applied to aged LCs to evaluate its biosafety. The results of light microscope observation and Annexin-V and PI staining and flow cytometry demonstrated that 100 mW/cm^2^ and 150 mW/cm^2^ LIPUS will cause much more apoptosis to LCs. Therefore, LIPUS lower than 100 mW/cm^2^ was safer for the stimulation of aged LCs in vitro (Figure 2A,B).

### 2.2. LIPUS Improves Proliferation and Secretion Function of Aged LCs 

**Figure 3:**
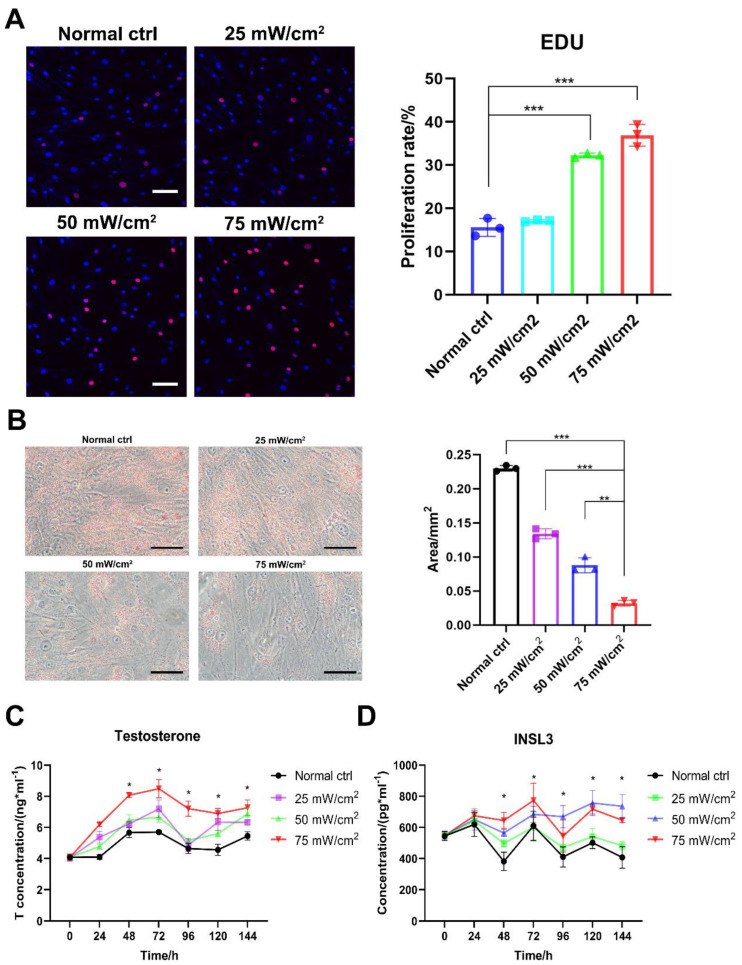
LIPUS improves proliferation and secretion function of aged LCs. LIPUS stimulation at 50 and 75 mW/cm^2^ promoted a significant increase in LCs proliferation compared with 25 mW/cm^2^ (**A**). Oil Red O staining showed a significant decrease in the amount of lipid droplets in LCs after LIPUS treatment of cells and an increase with higher energy (**B**). Testosterone and INSL3 levels were significantly higher in group treated with 50 mW/cm^2^ and 75 mW/cm^2^ after three consecutive days of LIPUS stimulation and following three days compared to the untreated group (**C**,**D**). The scale bar represents 100 μm. *** *p* < 0.001, ** *p* < 0.01, * *p* < 0.05.

Further observation of the effect of LIPUS treatment revealed that stimulation at 50 and 75 mW/cm^2^ promoted a significant increase in aged LCs proliferation compared with 25 mW/cm^2^ (Figure 3A). Oil Red O staining showed a significant decrease in the amount of lipid droplets in aged LCs after the LIPUS treatment of the cells and an increase with higher energy (Figure 3B). By assaying the testosterone and INSL3 levels at different time periods after LIPUS stimulation, we found that testosterone levels were significantly higher in the group treated with 50 mW/cm^2^ and 75 mW/cm^2^ after three consecutive days of LIPUS stimulation. Meanwhile, after three days of LIPUS stimulation, the in vitro testosterone production capacity of LCs was still significantly increased compared to the untreated group after the fluid change at the end of stimulation (Figure 3C,D). Also, testosterone and INSL3 secretion functions and intracellular lipid droplets are the functional features of LCs. Therefore, LIPUS treatments at 50 and 75 mW/cm^2^ were safe and effective for the stimulation of aged LCs.

### 2.3. LIPUS Delayed Cellular Senescence and Testosterone-Related Enzyme Synthesis Pathways

**Figure 4:**
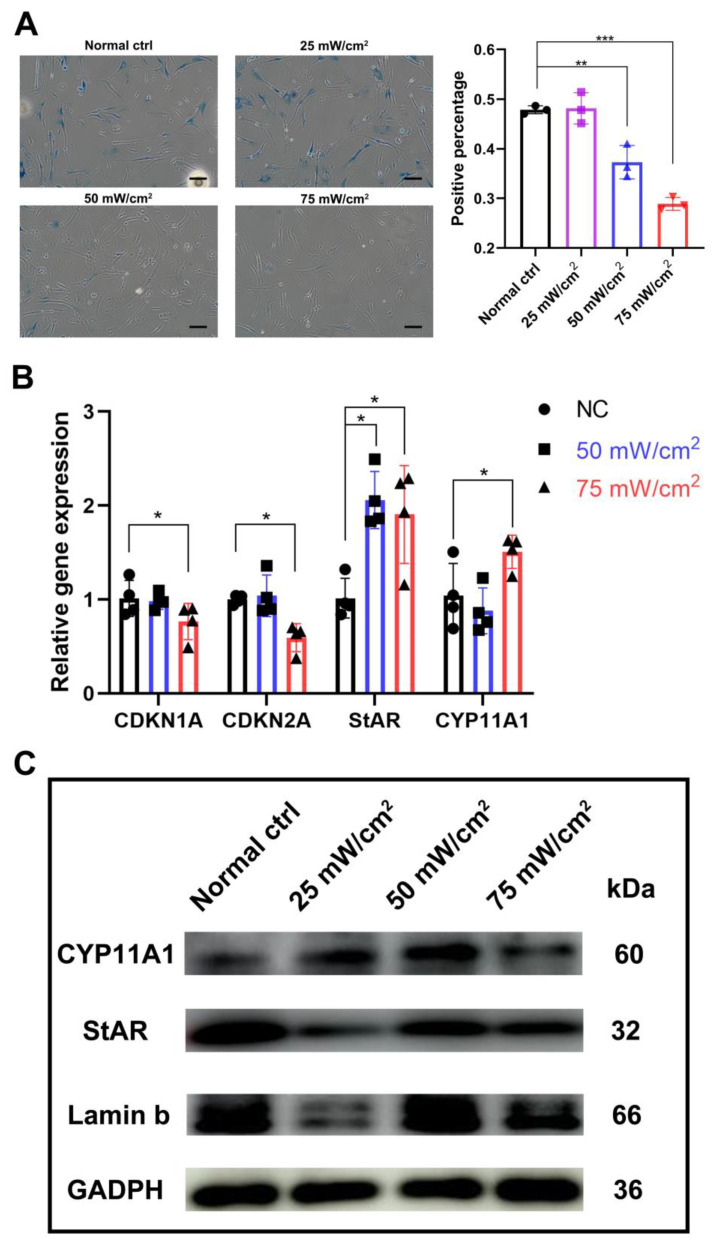
LIPUS delayed cellular senescence and testosterone-related enzyme synthesis pathways. (**A**) b-gal staining showed that the percentage of senescent cells was significantly lower in the 50 mW/cm^2^ and 75 mW/cm^2^ treated groups compared with untreated group, and the percentage was also significantly lower in the 75 mW/cm^2^ group compared to the 50 mW/cm^2^ group. (**B**) Transcript level change of aging-related genes CDKN1A(p21) and CDKN2A (p16) and testosterone synthesis pathway specific markers CYP11A1 and StAR. (**C**) Protein level changes of Lamin b, StAR and CYP11A1 after LIPUS treatment. The scale bar represents 100 μm. *** *p* < 0.001, ** *p* < 0.01, * *p* < 0.05.

After LIPUS treatment for 3 days, b-gal staining showed that the percentage of senescent cells was significantly lower in the 50 mW/cm^2^ and 75 mW/cm^2^ treated groups compared to the untreated group, and the percentage was also significantly lower in the 75 mW/cm^2^ group compared to the 50 mW/cm^2^ group (Figure 4A). The transcript levels of aging-related genes CDKN1A(p21) and CDKN2A (p16), and the protein levels of Lamin B were also examined; we found that the 75 mW/cm^2^ group reduced the expression of p16 and p21, while the 25 mW/cm^2^ group significantly lowered the Lamin b expression (Figure 4B,C and Figure S2). In addition, by analyzing the testosterone synthesis pathway specific markers CYP11A1 and StAR, we found that the expression level of StAR was significantly higher in both groups, while the level of CYP11A1 was significantly higher in the 75 mW/cm^2^ group compared to that of the untreated group and the 50 mW/cm^2^ group (Figure 4B). 

### 2.4. LIPUS Possess the Effect of Alleviating Leydig Cell Senescence and Testosterone Production Pathways

**Figure 5:**
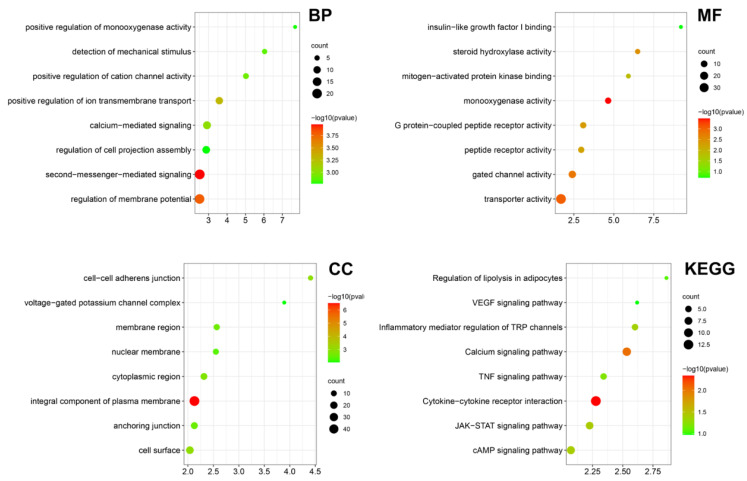
Transcriptome bioinformatic analysis of LCs with and without LIPUS stimulation. The Gene Ontology (GO) analysis including biological process (BP), cellular component (CC), and molecular function (MF) and Kyoto Encyclopedia of Genes and Genomes (KEGG) analysis according to the DEGs between the LIPUS group and normal control are shown as a bubble diagram. A gradient of red to green indicates low to high *p* values in the bubble diagram and the size of the bubbles indicates the count of enriched DEGs for each GO term.

Bulk RNA-seq was performed on the LIPUS treated testicular aged LCs in vitro, providing evidence for the effect and underlying mechanism of LIPUS. Then, we performed Gene Ontology (GO) and Kyoto Encyclopedia of Genes and Genomes (KEGG) analysis of DEGs between the LIPUS group and normal control. The main enriched GO terms in the biological process were “detection of mechanical stimulus”, “positive regulation of cation channel activity”, “positive regulation of ion transmembrane transport” and “second-messenger-mediated signaling”, suggesting that LIPUS activates the membrane ion channels. In the cellular component, the main enriched GO terms were “cell-cell adherens junction”, “voltage-gated potassium channel complex” and “membrane region”. While in molecular function, the main enriched GO terms were “insulin-like growth factor I binding”, “steroid hydroxylase activity”, “mitogen-activated protein kinase binding” and “monooxygenase activity”, indicating that LIPUS promoted Leydig cell proliferation and steroid hormone synthesis. In KEGG analysis, “Regulation of lipolysis in adipocytes”, “Inflammatory mediator regulation of TRP channels” and “TNF signaling pathway” were enriched (Figure 5). Together, these results indicated that LIPUS possesses the effect of alleviating Leydig cell senescence.

## 3. Discussion

Cellular senescence is a cell state implicated in various physiological processes and a wide spectrum of age-related diseases [15]. Declining testosterone status in aged men is one of the symptoms of the so-called late on set hypogonadism (LOH) resulting in significant detriment to the quality of life of older men [16,17]. Moreover, testicular aging is also accelerated by testis cancer and testis removal [18]. However, testosterone replacement therapy poses a potential risk to various organs including the prostate, testis and blood system as well as the cardiovascular and respiratory systems [19,20]. In this study, a potential new therapeutic approach for testicular senescence was explored by using LIPUS in vitro cellular assays.

Steroidogenesis involves mobilization of cholesterol from lipid droplets or plasma membranes, the transport of cholesterol to mitochondria, formation of pregnenolone in mitochondria, and subsequent conversion of pregnenolone to the final steroid product by enzymes of the smooth endoplasmic reticulum. [21]. 

Multiple defects have been identified in the steroidogenic pathway of aged LCs, including reductions in the cholesterol transport inducing protein steroidogenesis acute regulatory protein (StAR) and downstream steroidogenic enzymes of the mitochondria (CYP11A1) [22,23]. Our results showed that in vitro culture of aged LCs exhibited aging characteristics and decreased expression levels of the testosterone synthesis pathway compared to young LCs. After LIPUS treatment, StAR and CYP11A1 expression were significantly elevated, which explains the elevated testosterones, as well as the reduction in lipid droplets and increased cholesterol utilization after 75 mW/cm^2^ treatment, along with increased enzyme activity in the testosterone synthesis pathway, which enhances testosterone secretion. In addition, INSL3 is a peptide hormone secreted into the systemic circulation by LCs reflecting its number, functional capacity and extant differentiation status [24,25]. Histological studies of the human testes have also found that the LC numbers decreased during aging in parallel with reduced *INSL3* expression [5]. Our study also found that LIPUS enhanced the secretion of INSL3, which may be due to enhanced proliferation of LCs in vitro while reversing the senescence of LCs.

For cellular senescence, there are many reported drug treatments, including senescence secretome, mitochondrial and transcriptional regulators targeting [26,27,28]. However, the current treatment still has many side effects and has not been used for testicular LCs. The treatment of testicular aging is still dominated by testosterone replacement therapy, which has the disadvantage of increasing the risk of cardiovascular disease and prostate cancer, and causing the suppression of spermatogenesis [29,30,31]. It has been reported that the incidental risk of prostate cancer among other-based cancer surgery and androgen replacement therapy increases the risk of prostate cancer in these patients [32]. Meanwhile, targeted drug interventions and stem cells for aging have been reported in animal models, while the safety and efficacy of stem cell therapy need to be further confirmed in human LCs [33,34,35]. LIPUS is an appealing therapeutic option as it is a noninvasive treatment that has many advantages, including no risk of infection, no tissue damage and no known adverse reactions. Previous clinical studies of LIPUS have confirmed its biosafety in humans [36,37]. In this study, for the first time, human aged LCs were isolated and intervened with LIPUS in vitro and aged LCs has a senescence phenotype similar with what has been found in vivo, such as lipid droplet accumulation and decreased androgen and INSL3 levels [5,38,39]. After LIPUS treatment, testosterone secretion was enhanced similar with the results of previous in vivo studies in mice. Furthermore, the appropriate energy intensity for the in vitro treatment of human LCs was found to be lower than that previously used for spongiosa or other cells [36], probably due to differences in cell types and in vivo and in vitro energy transfer modes. At the same time, we also found that LCs showed many pathway changes after LIPUS stimulation, including the enrichment of lipid decomposition and steroid hydroxylase activity, which may be the direct reason for the reduction in lipid droplets and the increase in testosterone secretion after treatment. 

However, the present study still has some limitations. The therapeutic effect of LIPUS in LOH patients remains to be further confirmed and the risk of leydigioma needs be further verified in vivo. Moreover, the optimal treatment cycle and in vivo treatment intensity need to be further verified. In conclusion, this study established the safety and efficacy of LIPUS in the in vitro treatment of senescent LCs and confirmed its important role in reversing senescence and activating the testosterone synthesis pathway, which suggests that LIPUS is a potential non-invasive option for the treatment of LOH in aging men.

## 4. Materials and Methods

### 4.1. Donor Testicular Tissue

Human testicular tissues were retrieved from a donor 64-year-old patient (P21546) and 28-year-old patient (P22715); serum hormone levels and the hematoxylin–eosin (HE) staining of pathological section are shown in Supplementary Material and Table 1, as determined by histology after written informed consent at the Shanghai General Hospital. The specimens were immediately transported to the laboratory on ice and washed to remove any residual blood. This study was approved by the Ethical Review Committee of Shanghai General Hospital on 21 August 2020 (2020KY168).

### 4.2. Isolation and Immunofluorescence Staining of Human LCs

The procedure was followed as previously reported for the isolation of human LCs [13]. In detail, the fraction enriched for LCs was subjected to gravity sedimentation for 15 min. Then, cell suspensions were applied to a 4-layer discontinuous Percoll (GE Healthcare, Chicago, IL, USA) gradient of 21, 26, 34 and 60% in PBS-CMF. After centrifugation at 1500× *g* for 30 min, the cell bands from the second and third Percoll fraction (F2; between 26 and 34%) and F3 (between 34 and 60%) were collected separately, diluted with 2 volumes of DMEM:F12 with P/S, centrifuged at 200× *g* for 10 min and then plated and cultured in DMEM:F12 with P/S and 10% FBS. Cell culture media were removed and fresh media was added every 2–3 days. For immunocytochemistry staining, cells were fixed with 4% PFA for 30 min, washed three times with cold PBS (Gibco, New York, NY, USA), and permeabilized with 0.4% Triton X-100 (Sigma, St. Louis, MO, USA) for 5 min. After extensive wash with PBS, the cells were blocked in 5% bovine serum albumin (BSA, Sigma) for an hour at room temperature. The cells were then incubated with primary antibodies overnight at 4 °C at the following dilutions: rabbit polyclonal anti-CYP11A1 (dilution 1:200, Proteintech 13363-1-AP), mouse monoclonal anti-NES (dilution 1:200, Santa Cruz sc-23927) and mouse polyclonal anti-HSD3B (dilution 1:200, Santa Cruz sc-100466). Antigen detection was conducted using the appropriate combination of Alexa Fluor 488 and 594 secondary antibodies for 1 h at room temperature in the dark. Hoechst was used to label the nuclei. Images were captured with an OLYMPUS confocal microscope.

### 4.3. LIPUS Treatment

LIPUS treatment was applied to cultured LCs in vitro. Cell culture media were refreshed ahead of ultrasound exposure. A thin layer of ultrasound gel was applied to the LIPUS transducer before it was tightly appressed to the bottom of the culture plate. The cells were then exposed to ultrasound stimulation under the following conditions: a frequency of 1.7 MHz, a pulse duty cycle of 1: 4 (200 μs:800 μs), different energy intensities (25 mW/cm^2^, 50 mW/cm^2^, 75 mW/cm^2^, 100 mW/cm^2^, 150 mW/cm^2^) and an exposure time of 5 min. The treatment lasted for three days and the cell culture media were changed again at the end of the treatment.

### 4.4. Annexin-V and PI Staining and Flow Cytometry

Human Leydig cells were seeded at a density of 1 × 10^5^ cells/well in six-well plates in DMEM/F-12 supplemented with 10% FBS overnight. Cells were treated with LIPUS at levels of 0 mW/cm^2^, 25 mW/cm^2^, 50 mW/cm^2^, 75 mW/cm^2^, 100 mW/cm^2^ and 150 mW/cm^2^ for 5 min for 3 days and rested for 3 days. After 6 days, apoptosis percentages of human Leydig cells were detected using the Annexin V-FITC/PI Apoptosis Detection Kit by flow cytometry according to the manufacturer’s instructions (meilunbio MA0220). Cells were harvested and washed by cold PBS twice. Then the cells were resuspended by 1 × binding buffer to 1 × 10^6^ cells/mL. 5 μL of Annexin V-FITC and 7 μL of PI were added to each 100 μL of cell suspension and incubated for 15 min at room temperature. 400 μL of 1 × binding buffer were added and the stained cells were analyzed by flow cytometry.

### 4.5. Oil Red O Staining

Human Leydig cells were seeded at a density of 1 × 10^4^ cells/well in 35 mm glass bottom dishes with 20 mm micro-well in DMEM/F-12 supplemented with 10% FBS overnight. Cells were treated with LIPUS at level 0 mW/cm^2^, 25 mW/cm^2^, 50 mW/cm^2^, 75 mW/cm^2^ for 5 min for 3 days and rested for 3 days. After 6 days, the fat of human Leydig cells was detected using Oil Red O staining (Servicebio, G1015). Preparation of the working solution involved 6 parts of saturated Oil Red O staining solution and 4 parts of distilled water being mixed and incubated in 65 °C for 30 min. The cells were washed by PBS twice and fixed by 4% PFA. Then, 60% isopropyl alcohol was added to the well plate to cover the cells for 15–20 s. Oil Red O working solution was added to the well plate for 30 min at room temperature in the dark, then the staining solution was removed. Then, 60% isopropanol was added to the well plate for 3–5 s and washed with distilled water 3 times for 5 min each time. The cells were covered with PBS and observed under a microscope.

### 4.6. β-Gal Staining

Human Leydig cells were seeded at a density of 1 × 10^4^ cells/well in 35 mm glass bottom dishes with 20 mm micro-well in DMEM/F-12 supplemented with 10% FBS overnight. Cells were treated with LIPUS at levels 0 mW/cm^2^, 25 mW/cm^2^, 50 mW/cm^2^ and 75 mW/cm^2^ for 5 min for 3 days and rested for 3 days. After 6 days, β-galactosidase, a marker of senescence, was detected by β-Gal staining kit (ThermoFisher, Waltham, MA, USA, K146501). Cells were washed twice by PBS and fixed by fixative solution for 10 min at room temperature. After rinsing the plates twice with PBS, the staining solution was added to the plates for 2 h at 37 °C. The cells were checked under a microscope to count total cells and blue cells in 5–10 random fields of view.

### 4.7. RNA Extraction, RT-PCR, and Real-Time qPCR

Total RNA was extracted from the cultured cells or tissues using TRIzol (Takara, Kusatsu, Japan), and the quality and concentrations of total RNA were measured by NanoDrop (Thermo Fisher Scientific, Waltham, MA, USA). The ratio of A260/A280 of total RNA was set as 1.9~2.0 to ensure quality.

Reverse transcription (RT) of total RNA was conducted using the First Strand cDNA Synthesis Kit (Thermo Fisher Scientific, USA), and PCR of the cDNA was carried out according to the protocol as described previously. The primer sequences of chosen genes were designed and listed in Table S1. The PCR started at 94 °C for 2 min and was performed in terms of the following conditions: denaturation at 94 ℃ for 30 s, annealing at 55 ℃~60 ℃ for 45 s, and elongation at 72 ℃ for 45 s, for 35 cycles. The samples were incubated for an additional 5 min at 72 ℃. PCR with PBS but without cDNA served as a negative control. PCR products were separated by electrophoresis on 2% agarose gel and visualized with ethidium bromide. Images were recorded and band intensity was analyzed using chemiluminescence (Chemi-Doc XRS; Bio-Rad, Hercules, CA, USA).

### 4.8. ELISA

The hormone levels in the cell culture supernatants were measured using a commercially available ELISA kit (R&D) according to the manufacturer’s instructions. The absorbance at 450 nm was measured using an ELISA microtiter plate reader (Tecan, Mannedorf Switzerland). The hormone concentrations were evaluated according to a standard curve constructed by plotting the absorbance of each reference standard against its corresponding concentration. The coefficient of variation of this ELISA kit is 2.9–4% for intra-assay precision and 5.6–6.8% for inter-assay precision. The detectable dose of testosterone ranges from 0.012 to 0.041 ng/mL and the mean minimum detectable dose was 0.030 ng/mL.

### 4.9. RNA Sequencing

Human Leydig cells were seeded at a density of 5 × 10^5^ cells/well in 100 mm cell culture dishes in DMEM/F-12 supplemented with 10% FBS overnight. Cells were treated with LIPUS at levels of 0 mW/cm^2^ and 75 mW/cm^2^ for 5 min for 3 days and rested for 3 days. After 6 days, total RNA was extracted from the cultured cells using TRIzol reagent, and were subjected to sequencing (SINOTECH GENOMICS Shanghai, China). GO analysis was performed using WebGestalt (http://www.webgestalt.org/#, accessed on 21 October 2022).

### 4.10. Statistical Analyses

All data were analyzed using GraphPad Prism 6.0 (GraphPad Software) and results are presented as the mean ± standard deviation (SD). The Student’s *t*-test was used to analyze the differences between two groups. Analysis of variance (ANOVA) and a two-tailed *t*-test were used to analyze the differences between multiple groups. All the experiments were performed independently at least three times. All statistical tests were two-tailed and *p* value < 0.05 was considered a statistically significant difference.

## Figures and Tables

**Table 1:** Donor age and hormone information.

	Old	Young
Age	64	28
Estradiol	25.00 pg/mg	27.00 pg/mg
LH	11.17 IU/L	3.57 IU/L
FSH	12.43 IU/L	2.10 IU/L
Prolactin	1.76 ng/ml	7.58 ng/ml
Progesterone	<0.08 ug/L	0.25 ug/L
Testosterone	1.34 ug/L	5.76 ug/L

## Data Availability

The data that support the findings of this study are available on request from the corresponding author.

## Supplementary Materials

The following supporting information can be downloaded at: https://www.mdpi.com/article/10.3390/ijms24010418/s1.
