# Supplementary material for: Low-Intensity Pulsed Ultrasound Alleviates Human Testicular Leydig Cell Senescence In Vitro
Source: Int J Mol Sci. 2022 Dec 27;24(1):418. doi: 10.3390/ijms24010418 (PMC9820771; doi:10.3390/ijms24010418)
Supplement: Supplementary file 1 [file ijms-24-00418-s001.zip › ijms-2032927-supplementary.pdf]

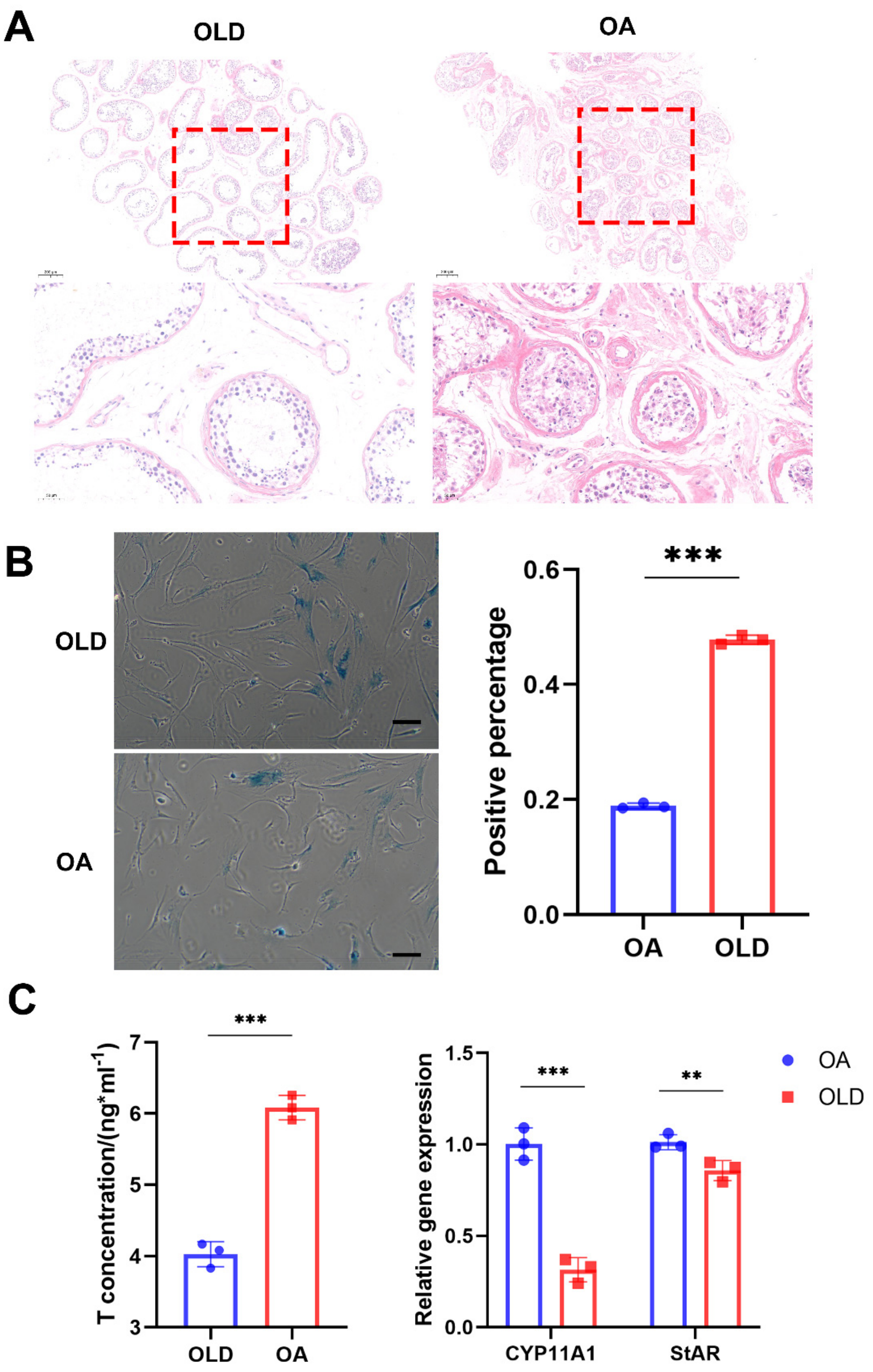


Figure S1. HE pathological section and comparison of young and old LC differences. (A) HE staining of donor testes (B) b-gal staining of LCs showing significantly higher percentage of positives in older men (C) Significantly lower levels of testosterone secretion and transcript levels of key factors of the testosterone synthesis pathway in older LCs in vitro. The scale bar represents 100 μm. *** $p < 0.001$, ** $p < 0.01$, * $p < 0.05$.

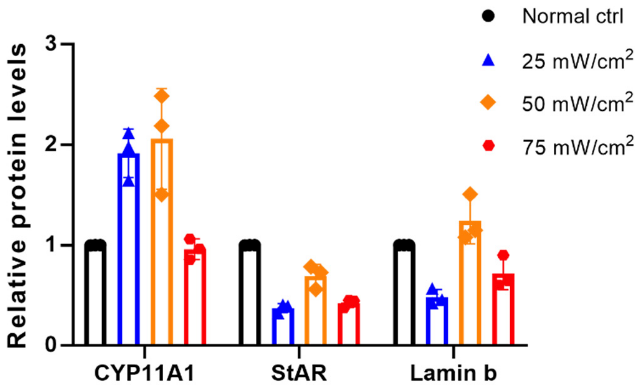


Figure S2. Densitometry readings/intensity ratio of each band of WESTERN BLOT.

Table S1. PCR PRIMERS

| | |
|---|---|
| CDKN2A-F | CCGAATAGTTACGGTCGGAGG |
| CDKN2A-R | CACCAGCGTGTCCAGGAAG |
| CDKN1A-F | AGTCAGTTCCTTGTGGAGCC |
| CDKN1A-R | CATTAGCGCATCACAGTCGC |
| StAR-F | GGCTACTCAGCATCGACCTC |
| StAR-R | CTAAACACGAACCCCACCCA |
| CYP11A1-F | CCAGACCTTTCTGAGTGCCC |
| CYP11A1-R | CTCCCTGTAAATCGGGCCAT |
